# Association of Chlorhexidine Use and Scaling and Root Planing With Birth Outcomes in Pregnant Individuals With Periodontitis: A Systematic Review and Meta-analysis

**DOI:** 10.1001/jamanetworkopen.2022.47632

**Published:** 2022-12-19

**Authors:** Anwar T. Merchant, Rajat Das Gupta, Maxwell Akonde, Mark Reynolds, Stephanie Smith-Warner, Jihong Liu, Fouzia Tarannum, James Beck, Donald Mattison

**Affiliations:** 1 Department of Epidemiology and Biostatistics, Arnold School of Public Health, University of South Carolina, Columbia; 2 School of Dentistry, University of Maryland Baltimore, Baltimore; 3 Department of Epidemiology, Harvard T.H. Chan School of Public Health, Boston, Massachusetts; 4 M. R. Ambedkar Dental College and Hospital, Bangalore, India; 5 Division of Comprehensive Oral Health and Periodontology, University of North Carolina, Chapel Hill

## Abstract

**Importance:**

Chlorhexidine mouthwash enhances treatment effects of conventional periodontal treatment, but data on chlorhexidine as a source of heterogeneity in meta-analyses assessing the treatment of maternal periodontitis in association with birth outcomes are lacking.

**Objective:**

To assess possible heterogeneity by chlorhexidine use in randomized clinical trials (RCTs) evaluating the effect of periodontal treatment (ie, scaling and root planing [SRP]) vs no treatment on birth outcomes.

**Data Sources:**

Cochrane Oral Health’s Trials Register, Cochrane Pregnancy and Childbirth’s Trials Register, Cochrane Central Register of Controlled Trials (CENTRAL), MEDLINE Ovid, Embase Ovid, LILACS BIREME Virtual Health Library (Latin American and Caribbean Health Science Information database), US National Institutes of Health Ongoing Trials Register (ClinicalTrials.gov), and the WHO International Clinical Trials Registry Platform were searched through March 2022.

**Study Selection:**

RCTs were included if they were conducted among pregnant individuals with periodontitis, used interventions consisting of SRP vs no periodontal treatment, and assessed birth outcomes.

**Data Extraction and Synthesis:**

Data were abstracted with consensus of 2 reviewers using Rayyan and assessed for bias with the Cochrane Risk of Bias 2 tool before random effects subgroup meta-analyses. Analyses were conducted following the Preferred Reporting Items for Systematic Reviews and Meta-analyses reporting guideline.

**Main Outcomes and Measures:**

Outcomes of interest were preterm birth (ie, <37 weeks’ gestation) and low birth weight (ie, <2500 g).

**Results:**

There were 12 studies with a total of 5735 participants evaluating preterm birth. Control group participants did not receive any treatment or use chlorhexidine during pregnancy. All intervention group participants received SRP; in 5 of these studies (with 2570 participants), pregnant participants in the treatment group either received chlorhexidine mouthwash or advice to use it, but participants in the remaining 7 studies (with 3183 participants) did not. There were 8 studies with a total of 3510 participants evaluating low birth weight, including 3 studies with SRP plus chlorhexidine (with 594 participants) and 6 studies with SRP only (with 2916 participants). The SRP plus chlorhexidine groups had lower risk of preterm birth (relative risk [RR], 0.56; 95% CI, 0.34-0.93) and low birth weight (RR, 0.47; 95% CI, 0.32-0.68) but not the SRP-only groups (preterm birth: RR, 1.03; 95% CI, 0.82-1.29; low birth weight: RR, 0.82; 95% CI, 0.62-1.08).

**Conclusions and Relevance:**

These findings suggest that treating maternal periodontitis with chlorhexidine mouthwash plus SRP was associated with reduced risk of preterm and low birth weight. Well-conducted RCTs are needed to test this hypothesis.

## Key Points

**Question:**

Is treating pregnant individuals with periodontitis with chlorhexidine mouthwash plus scaling and root planing associated with birth outcomes?

**Findings:**

In this systematic review and meta-analysis including 12 studies with 5735 participants, chlorhexidine mouthwash plus scaling and root planing, but not scaling and root planing alone, was associated with reduced risks of preterm birth and low birth weight.

**Meaning:**

These findings suggest that using chlorhexidine mouthwash with scaling and root planing to treat periodontitis in pregnant individuals may improve birth outcomes.

## Teaser

This systematic review and meta-analysis assesses heterogeneity by chlorhexidine use in randomized clinical trials evaluating the effect of scaling and root planing (SRP) vs no treatment on birth outcomes.

## Introduction

Although maternal periodontitis is associated with increased risk of adverse birth outcomes in observational studies^1,2,3^ and results from microbiological and animal studies support its biologic plausibility,^4,5,6^ evidence from randomized clinical trials (RCTs) evaluating periodontal treatment in relation to birth outcomes is equivocal.^7^ A Cochrane review and meta-analysis evaluating 11 RCTs^7^ comparing periodontal treatment with no treatment during pregnancy reported no clear difference in preterm birth at less than 37 weeks’ gestation but did find a reduction in incidence of low birth weight (ie, <2500 g) favoring treatment.^7^ The review assessed the overall quality of evidence to be low and attributed it largely to lack of blinded treatment, ineffective randomization in some studies, and heterogeneity in populations and treatment protocols. The effect of periodontal treatment on birth outcomes could also have been underestimated in intention-to-treat analyses of RCTs if the intervention benefited both fetal survival and the outcomes when outcomes were determined only if the fetus survived (such as the case in preterm birth or low birth weight).^8,9,10^

Five of the studies included in the Cochrane review used chlorhexidine mouthwash in addition to scaling and root planing (SRP) in the intervention groups.^11,12,13,14,15^ Chlorhexidine mouthwash used in conjunction with SRP among individuals with periodontal disease has been reported to be associated with reduced dental plaque, mild gum disease, gum bleeding,^16^ and counts of *Fusobacterium nucleatum*,^17^ an organism that has been associated with both periodontal disease and adverse birth outcomes.^18^ Chlorhexidine and cetylpyridium chloride mouthwashes disrupt oral biofilm formation and maturation, thereby reducing the bacterial bioburden in healthy adults without periodontitis.^19^ Cetylpyridinium chloride mouthwash, which has a similar effect on the oral microbiome as chlorhexidine, improved the oral health of pregnant participants and reduced risk of premature rupture of membranes in one RCT,^20^ and reduced risk of preterm birth by 74% in another.^21^ Chlorhexidine has been recommended in low-income settings for intrapartum and vaginal wiping, neonatal wiping, and umbilical cord cleaning during childbirth to improve birth outcomes.^22^ Therefore, chlorhexidine mouthwash use could be a source of heterogeneity in the Cochrane review.^7^

To test this hypothesis, we updated a recently conducted Cochrane review^7^ of RCTs evaluating SRP in relation to birth outcomes, classifying the studies by whether the intervention additionally included chlorhexidine mouthwash. We then conducted a subgroup meta-analysis by chlorhexidine use.

## Methods

We submitted the protocol to PROSPERO (ID No. CRD42022307420). The study was conducted following the Preferred Reporting Items for Systematic Reviews and Meta-analyses (PRISMA) reporting guideline.

### Eligibility Criteria

For this systematic review and meta-analysis, RCTs were included if they were conducted among pregnant participants with periodontitis, used interventions consisting of mechanical periodontal treatment (ie, SRP) with or without chlorhexidine mouthwash vs no periodontal treatment or mouthwash use during pregnancy, ascertained birth outcomes, and reported preterm birth (ie, <37 weeks’ gestation) or low birth weight (ie, <2500 g). RCTs evaluating the effect of mouthwash alone on birth outcomes were not included.

### Information Sources and Search Strategy

We included all the studies in the 2017 Cochrane review on this topic conducted by Iheozor-Ejiofor and colleagues^7^ and updated the search using the search criteria in that report from October 2016 through March 2022. With the help of a medical librarian, we conducted the search in the Cochrane Oral Health’s Trials Register, Cochrane Pregnancy and Childbirth’s Trials Register, Cochrane Central Register of Controlled Trials (CENTRAL), MEDLINE Ovid, Embase Ovid, LILACS BIREME Virtual Health Library (Latin American and Caribbean Health Science Information database), US National Institutes of Health Ongoing Trials Register (ClinicalTrials.gov), and the WHO International Clinical Trials Registry Platform. Details of the search terms and strategy are provided in the eAppendix in Supplement 1.

### Selection Process

Search results were entered into Rayyan software.^23^ After removing duplicates, 2 reviewers (R.D.G. and M.A.) independently reviewed the titles and abstracts using prespecified criteria and selected full articles for review. The reviewers sorted out disagreements on study selection through discussion. The final set of studies to be included in the meta-analysis were evaluated for risk of bias.

### Data Collection

The number of events and total number of participants by intervention group (SRP with or without chlorhexidine mouthwash vs no treatment or mouthwash use) were entered into Excel (2010 release) spreadsheets (Microsoft) for preterm birth and low birth weight separately. We also collected information about chlorhexidine use and year of study. The studies conducted by López and colleagues^11,12^ excluded individuals who were lost to follow-up and preterm births that were planned. We included all participants who were randomized and spontaneous or planned preterm births in the López studies,^11,12^ while the Cochrane review^7^ included just unplanned preterm births reported in the López et al studies.

### Risk of Bias

Study quality was determined by the Cochrane Risk of Bias 2 tool. Two investigators (A.T.M. and R.D.G.) independently reviewed all 12 studies for bias using the Risk of Bias 2 template and arrived at a single measure following discussion.

### Statistical Analysis

Log risk ratios of the effect sizes were summarized with random-effects models using Stata software version 17 (StataCorp). Subgroup analyses by chlorhexidine use were conducted. The log risk ratios and summary measures were exponentiated before displaying the results in forest plots. Publication bias was assessed using a funnel plot. The risk of bias plot was prepared using R statistical software version 4.2.1 (R Project for Statistical Computing). We used the *Q*-statistic with a 2-sided *P* < .05 to evaluate a common association across the studies.

We repeated the analyses using the preterm definitions in the Cochrane review. To evaluate the excess influence of any one study we repeated the main analyses for preterm with Stata’s leave-one-out option. Data were analyzed from May 18 to August 25, 2022.

## Results

**Figure 1.**
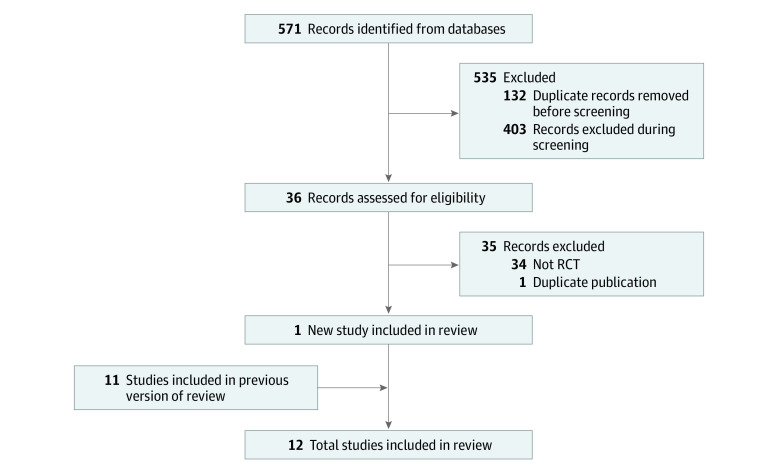
Flow Diagram for Updated Systematic Review RCT indicates randomized clinical trial.

The systematic search had 571 results consisting of 439 unique records; after reviewing titles and abstracts, 36 full papers were reviewed, yielding 1 new study, which was included in this update (Figure 1). Thus, this systematic review and meta-analysis included 12 studies^7,11,12,13,14,15,24,25,26,27,28,29^ with 5735 participants evaluating preterm birth (<37 weeks); chlorhexidine mouthwash was used in 5 of those studies^11,12,13,14,15^ (with 2570 participants) and not in 7 studies^7,24,25,26,27,28,29^ (with 3183 participants). There were 8 studies with 3510 participants evaluating low birth weight (<2500 g), including 3 studies^12,13,14^ that used chlorhexidine (with 594 participants) and 5 studies^24,26,27,28,29^ that did not (2916 participants). Details of the studies are described in the Table. The risk of bias assessments are presented in eFigure 1 in Supplement 1, and funnel plot are presented in eFigure 2 in Supplement 1. Bias was likely in most of the studies, largely resulting from deviations from the intervention (eFigure 1 in Supplement 1). The point estimates of effect measures of 2 studies^14,29^ that were consistent with a strong protective effect had the larger standard errors (eFigure 2 in Supplement 1).

**Table.** Description of Studies Included in the Systematic Review and Meta-analysis

Source	Country	Participants, No.	Age, mean (SD), y	Intervention	Control	Chlorhexidine	Duration of pregnancy measured	Birth weight measured
Farrell et al,^7^ 2003	United Kingdom	198	NR	Oral hygiene instructions, SRP, follow-up at 30 wk, maintenance monthly	Option for dental treatment after birth	No	Started at GA 12 wk	Measured but LBW was NR
Herera et al,^25^ 2009	Colombia	60 (mild preeclampsia)	Intervention: 24 (6.5); control: 27 (7.6)	Oral hygiene instructions, SRP, single session performed at GA 26-34 wk	Conventional medical treatment (procedure NR)	No	Mean GA, 31.8 wk	Percentile weight adjusted to GA
López et al,^12^ 2002	Chile	400 (49 excluded)	Intervention: 28 (4.5);control: 27 (4.3)	Oral hygiene instructions, SRP, maintenance therapy every 2-3 wk until delivery, daily rinsing with 0.12% chlorhexidine	Oral health monitored every 4-6 wk during the gestational period	Yes	Preterm: <37 wk; term: ≥37 wk	LBW, defined as <2500 g
López et al,^11^ 2005	Chile	870 (36 excluded)	Intervention: 25.5 (5.4); control: 25.0 (4.6)	Oral hygiene instructions, SRP, maintenance therapy every 2-3 wk until delivery, daily rinsing with 0.12% chlorhexidine	Monitored 2-3 times during the pregnancy	Yes	Preterm: <37 wk; term: ≥37 wk	LBW, defined as <2500 g
Michalowicz et al,^24^ 2006	United States	823	Intervention: 26.1 (5.6); control: 25.9 (5.5)	Oral hygiene instruction, SRP up to 4 visits, monthly tooth polishing until delivery	Oral examination at monthly follow-ups, same number of these visits as the treatment group	No	Preterm: <37 wk; term: ≥37 wk	Weight in grams and grouped as <2500 g for LBW
Newnham et al,^15^ 2009	Australia	1082 (542 for treatment and 540 for control)	Both: 30.5 (5.5)	Oral hygiene instructions, SRP, maintenance therapy every 2-3 wk until delivery, advised daily rinsing with 0.12% chlorhexidine mouthwash	Treatment 6 wk after delivery	Yes	GA	Continuous
Offenbacher et al,^26^ 2009	United States	1806	Intervention: 25.3 (5.5); control: 25.4 (5.5)	Oral hygiene instructions, SRP, up to 4 sessions	Treatment after delivery	No	Preterm: <37 wk; term: ≥37 wk	Birth weight <2500 g and <1500 g
Olivera et al,^27^ 2011	Brazil	246	Intervention: 30.0 (4.4); control: 26.6 (4.0)	Oral hygiene instructions, SRP, periodontal maintenance visits every 3 wk	Treatment after delivery, reexamined during the 30th and 32nd weeks’ GA	No	Preterm: <37 wk; term: ≥37 wk	LBW, defined as <2500 g
Radnai et al,^28^ 2009	Hungary	83	Intervention: 29.1 (6.4); control: 28.9 (5.4)	Oral hygiene instruction, SRP	No treatment during pregnancy	No	Preterm: <37 wk; term: ≥37 wk	LBW, defined as <2500 g
Sadatmansouri et al,^14^ 2006	Iran	30	Intervention: 29.1 (4.3); control: 28.4 (4.1)	SRP and use of 0.2% chlorhexidine mouth rinse for 1 wk	No treatment during pregnancy	Yes	Preterm: <37 wk; term: ≥37 wk	Preterm LBW, defined as <2500 g
Tarannum et al,^13^ 2007	India	200	Intervention: 23 (3.3); control: 22.9 (3.6)	Oral hygiene instruction, SRP, rinsing twice daily with 0.2% chlorhexidine, 4-5 weekly appointments, periodontal maintenance every 3-4 wk until delivery	No treatment during pregnancy	Yes	Preterm: <37 wk; term: ≥37 wk	LBW, defined as <2500 g
Caneiro-Queija et al,^29^ 2019	Spain	40	Intervention: 32.0 (4.3); control: 32.3 (4.1)	Oral hygiene instructions, SRP	Oral hygiene instructions, professional tooth cleaning	No	Preterm: <37 wk; term: ≥37 wk	LBW, defined as <2500 g

Abbreviations: GA, gestational age; LBW, low birth weight; NR, not reported; SRP, scaling and root planning.

**Figure 2.**
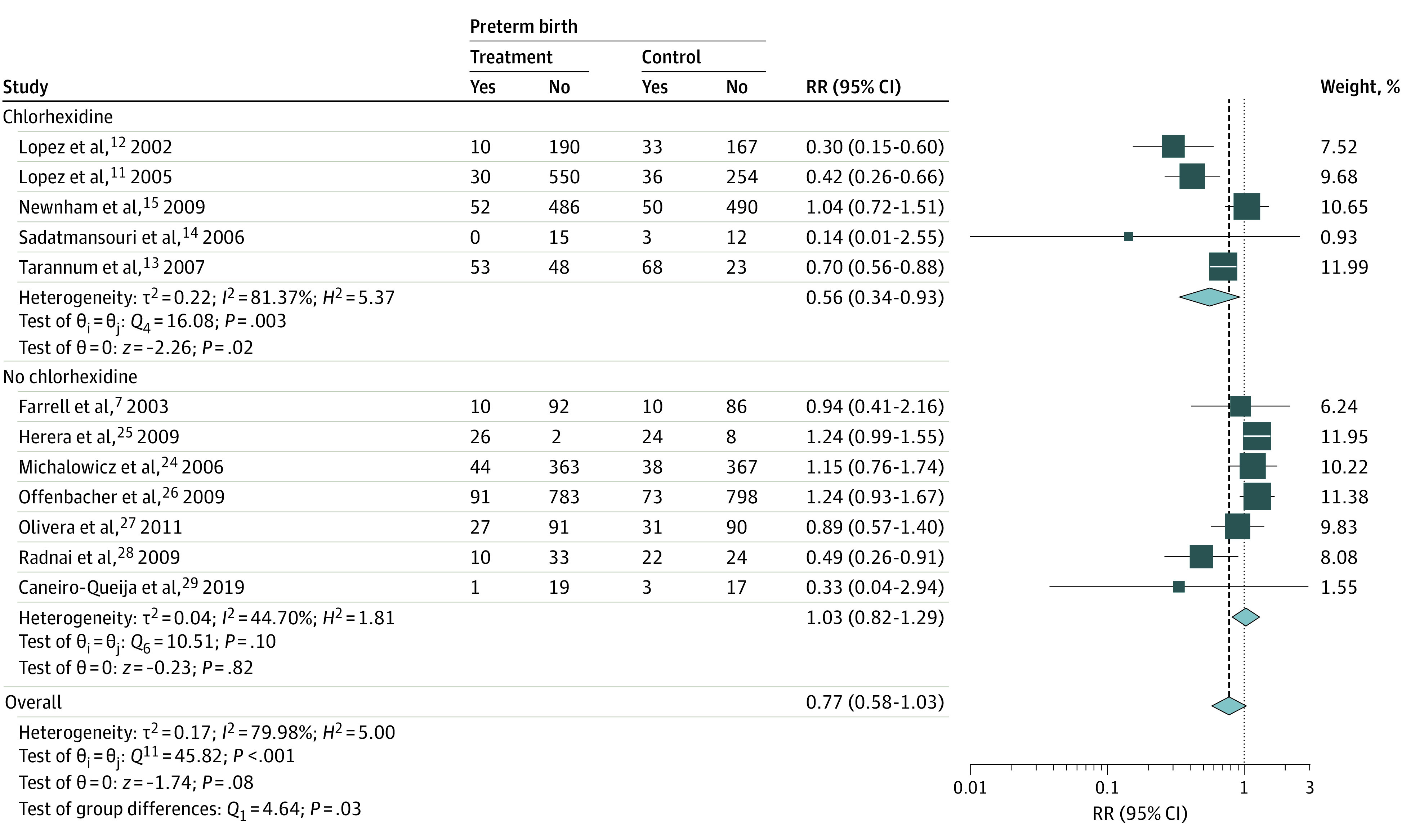
Association Between Scaling and Root Planing With and Without Chlorhexidine Mouthwash and Preterm Birth in Mothers With Periodontitis Squares indicate relative risk (RR), with size of squares indicating weight; horizontal lines, 95% CIs for the RRs; diamond, pooled estimate, with points of the diamond indicating the 95% CI for the pooled estimate.

Periodontal treatment was not associated with preterm birth in the combined estimate from all 12 RCTs (relative risk [RR], 0.77, 95% CI, 0.58-1.03); however, there was evidence of heterogeneity across the studies (*Q*_11_ = 45.82; *P* < .001). In subgroup analyses, a lower risk of preterm birth was observed when chlorhexidine was added to the treatment of maternal periodontitis (RR, 0.56; 95% CI, 0.34-0.93), but there was no association when chlorhexidine was not included in the treatment (RR, 1.03; 95% CI, 0.82-1.29). The risk estimates in the subgroups were different (between-group *Q*_1_ = 4.64; *P* = .03); there was evidence of heterogeneity within the chlorhexidine subgroup (*Q*_4_ = 16.08; *P* < .001) but not in the no-chlorhexidine subgroup (*Q*_6_ = 10.51; *P* = .10) (Figure 2).

**Figure 3.**
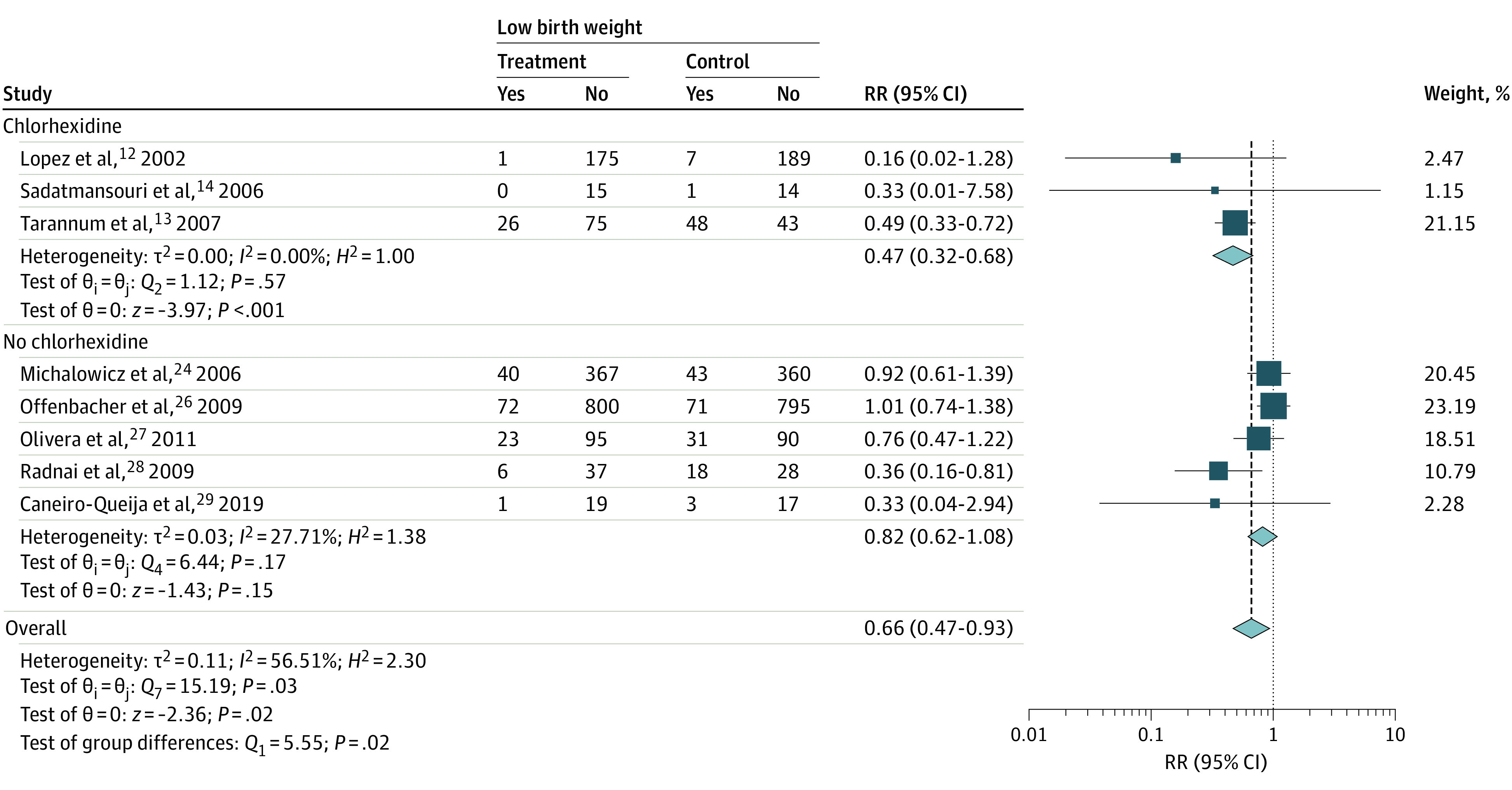
Association Between Root Planing With and Without Chlorhexidine Mouthwash and Low Birth Weight in Mothers With Periodontitis Squares indicate relative risk (RR), with size of squares indicating weight; horizontal lines, 95% CIs for the RRs; diamond, pooled estimate, with points of the diamond indicating the 95% CI for the pooled estimate.

Periodontal treatment was associated with low birth weight in the overall estimate from 8 RCTs (RR, 0.66; 95% CI, 0.47-0.93), but there was evidence of heterogeneity across the studies (*Q*_7_ = 15.19; *P* = .03). Periodontal treatment was associated with lower risk of low birth weight when chlorhexidine was included (RR, 0.47; 95% CI, 0.32-0.68) but not alone (RR, 0.82; 95% CI, 0.62-1.08). There was no evidence of heterogeneity within the subgroups (chlorhexidine group: *Q*_2_ = 1.12; *P* = .57; no-chlorhexidine group: *Q*_4_ = 6.44; *P* = .17). The test of difference in the chlorhexidine vs no chlorhexidine subgroups was *Q*_1_) = 5.55 (*P* = .02) (Figure 3).

Results were qualitatively similar to those in this report when the data were reanalyzed using the preterm definition for the López et al studies^11,12^ as in the Cochrane review (eFigure 3 in Supplement 1). The effect estimates were qualitatively similar in the leave-one-out analyses for preterm birth (eFigure 4 in Supplement 1). The number of studies was too small to conduct similar analyses for low birth weight.

## Discussion

In this systematic review and meta-analysis of 12 RCTs^7,11,12,13,14,15,24,25,26,27,28,29^ including 5735 participants, prenatal periodontitis treated with chlorhexidine mouthwash plus periodontal treatment was associated with lower risk of preterm birth and low birth weight compared with the control group consisting of pregnant participants who did not receive periodontal treatment or use chlorhexidine mouthwash during pregnancy. Periodontal treatment without concomitant chlorhexidine mouthwash use was not associated with birth outcomes.

Our analysis is an update of a 2017 Cochrane review^7^ of 11 RCTs that summarized the association between treating maternal periodontitis and birth outcomes. That review^7^ reported that the intervention was not associated with preterm birth but had a beneficial association with reduced incidence of low birth weight, but that the RCTs in the review were of low quality and heterogeneous. A critical difference between the 2017 Cochrane review^7^ and our analyses is that in the Cochrane review, interventions consisting of periodontal treatment with and without chlorhexidine were combined. In this study, we examined these studies separately. We found that periodontal treatment showed beneficial associations with preterm birth and low birth weight only when combined with chlorhexidine mouthwash. One explanation for these results is bias,^7^ which was judged to be present in the studies that used chlorhexidine and was attributed to unclear allocation concealment,^11,12,13^ lack of blinding in outcome assessment,^13^ incomplete outcome data,^12^ selective reporting,^13^ and imbalance of participant characteristics across treatment groups.^11,12^ However, bias was present to varying degrees in the other studies included in the Cochrane review.^7^ For example, periodontal status worsened in the treatment and control groups at 2 of 3 sites in the study conducted by Offenbacher and colleagues,^30^ indicating that periodontal treatment was probably ineffective at controlling oral infection.

### Limitations

This study has some limitations. One limitation was that all the RCTs included in this analysis were susceptible to survival bias, which occurs if the intervention benefits both the outcome and fetal survival.^8,31^ Survival bias could have underestimated a potentially beneficial treatment effect in RCTs evaluating treatment of maternal periodontal disease in relation to birth outcomes. For example, in the Obstetrics and Periodontal Therapy study,^24^ treatment and control groups were evenly balanced at the start of the study, but during follow-up, there were more fetal losses in the control group than the intervention group (3.4% vs 1.2%). After correction for bias, the intention-to-treat analyses changed from an odds ratio of 1.14 (95% CI, 0.72-1.81) before correction to 0.94 (95% CI, 0.48-1.82).^9^ In another large study evaluating this question among 1078 participants, there were 4 stillbirths during follow-up, all of which were in the control group.^15^ The probability of this happening by chance is 0.0625. We were unable to correct for this bias because we did not have access to the primary data for the study. In both of these studies, the investigators pointed out these potential limitations, but bias correction was not done in these or any of the other studies, which could have underestimated any potentially beneficial effect of the intervention.

Another limitation was that adherence with chlorhexidine mouthwash was not reported in any of the studies. In 3 of 5 studies evaluating preterm birth, the investigators provided the participants chlorhexidine mouthwash, while in the other 2, participants were advised to use it. This could be a source of heterogeneity in the studies that used chlorhexidine. Another methodologic shortcoming of the RCTs was that they varied in size and were too small for assessment of potential effect modification by other factors, such as oral health status at baseline, which could help identify participants most likely to benefit from the intervention if it was indeed effective. Apart from the study conducted by Newnham and colleagues^15^ in Australia among mostly well-educated White participants with possibly less severe periodontal disease, the remaining 4 studies that used chlorhexidine mouthwash were conducted in less affluent communities among participants with poor oral health, possibly contributing to heterogeneity. Indeed, such observations have been interpreted to suggest that individuals at higher risk of adverse pregnancy outcomes due to income inequality and racial and ethnic disparities^32^ or residence in low-resource settings^33^ may differentially benefit more from interventions targeting reductions in oral infection and inflammation.^34,35^

It is plausible that beneficial effects of chlorhexidine on the microbiome, oral health, and on the effectiveness of SRP may also account, at least partially, for the differences in results between studies including chlorhexidine and those that did not. *F nucleatum* is associated not only with periodontitis prevalence^36^ and progression,^37^ but also with chorioamnionitis,^38,39^ preterm birth,^40,41^ stillbirth,^42^ neonatal sepsis,^43^ and preeclamplia.^44^ In adverse pregnancy outcomes, *F nucleatum* has been isolated from the amniotic sac, fetal membranes, cord blood, fetal lung, neonatal gastric aspirates, and stomach.^38,39,41,42,43,45,46,47,48^ Infective strains of *F nucleatum* produce protein adhesion A, which binds to vascular endothelial cadherin, increasing endothelium permeability, allowing *F nucleatum* and other organisms to enter the systemic circulation.^49^ In the placenta, *F nucleatum* with protein adhesion A increases permeability of the vascular endothelium of the umbilical blood vessels, making a pathway for it to enter the amniotic sac.^50^ In a double-blind placebo-controlled RCT, an intervention consisting of chlorhexidine and cetylpyridinium chloride mouthwash reduced dental plaque, gingival bleeding, and counts of *F nucleatum* and *Prevotella intermedia* in 3 months among individuals with moderate to severe periodontitis who were unable to maintain good oral hygiene.^17^ A Cochrane review^16^ of RCTs evaluating chlorhexidine mouthwash as an adjunct to mechanical oral hygiene found that chlorhexidine use was associated with less dental plaque, gingival bleeding, and gingival inflammation over 4 to 6 weeks among individuals with periodontal disease. In a crossover trial, chlorhexidine mouthwash increased abundance of *Firmicutes* and *Proteobacteria* (associated with good oral health) and reduced content of *Bacteroidetes,* Saccharibacteria*, SR1*, and *Fusobacteria* (associated with poor oral health) among individuals without periodontal disease.^19^ Cetylpyridinium chloride mouthwash without mechanical oral hygiene reduced preterm birth in pregnant individuals with periodontal disease who were at high risk of preterm birth,^21^ and it reduced risk of premature rupture of membranes without affecting preterm birth in another RCT.^20^ Thus, the addition of an antimicrobial mouthwash, such as chlorhexidine, could enhance the protective effect of SRP in adverse birth outcomes. This may explain why conventional periodontal treatment was associated with favorable birth outcomes when combined with chlorhexidine use but not otherwise. Despite weaknesses in the RCTs included in this review, it is possible that a true causal effect may also be present.

## Conclusions

The findings of this systematic review and meta-analysis are consistent with the hypothesis that adding chlorhexidine to conventional treatment of maternal periodontitis has a protective association in preventing adverse birth outcomes. The risks of preterm birth and low birth weight were lower in the subgroups in which chlorhexidine mouthwash was added to conventional periodontal treatment vs when it was not.

A large, well-conducted RCT evaluating a combination of antimicrobial mouthwash and periodontal treatment in relation to birth outcomes would answer this question. However, such a study has not been conducted, to our knowledge. Our findings, taken together with emerging evidence of the role of *F nucleatum* in preterm birth and the effectiveness of chlorhexidine, suggest that treating maternal periodontal disease with a combination of an antimicrobial mouthwash and conventional periodontal treatment may improve birth outcomes.

## Notes

Supplement 1. **eAppendix.** Search Strategy for the Systematic Review and Meta-analysis **eFigure 1.** Risk of Bias Chart Using the ROB 2 Tool **eFigure 2.** Funnel Plot of Studies Included in Systematic Review and Meta-analysis **eFigure 3.** Analyses for Preterm Birth Repeated Using Definition in Cochrane Review **eFigure 4.** Leave-One-Out Meta-analyses for Preterm Birth

Supplement 2.
Data Sharing Statement
