# Supplementary material for: Association of Chlorhexidine Use and Scaling and Root Planing With Birth Outcomes in Pregnant Individuals With Periodontitis: A Systematic Review and Meta-analysis
Source: JAMA Netw Open. 2022 Dec 19;5(12):e2247632. doi: 10.1001/jamanetworkopen.2022.47632 (PMC9856591; doi:10.1001/jamanetworkopen.2022.47632)
Supplement: Supplement 1. — eAppendix. Search Strategy for the Systematic Review and Meta-analysis eFigure 1. Risk of Bias Chart Using the ROB 2 Tool eFigure 2. Funnel Plot of Studies Included in Systematic Review and Meta-analysis eFigure 3. Analyses for Preterm Birth Repeated Using Definition in Cochrane Review eFigure 4. Leave-One-Out Meta-analyses for Preterm Birth [file jamanetwopen-e2247632-s001.pdf]

Supplemental Online Content

Merchant AT, Gupta RD, Akonde M, et al. Association of chlorhexidine use and scaling and root planing with birth outcomes in pregnant individuals with periodontitis: a systematic review and meta-analysis. *JAMA Netw Open*. 2022;5(12):e2247632. doi:10.1001/jamanetworkopen.2022.47632

eAppendix. Search Strategy for the Systematic Review and Meta-analysis

eFigure 1. Risk of Bias Chart Using the ROB 2 Tool

eFigure 2. Funnel Plot of Studies Included in Systematic Review and Meta-analysis

eFigure 3. Analyses for Preterm Birth Repeated Using Definition in Cochrane Review

eFigure 4. Leave-One-Out Meta-analyses for Preterm Birth

This supplemental material has been provided by the authors to give readers additional information about their work.

© 2022 Merchant AT et al. *JAMA Network Open.*

eAppendix. Search Strategy for the Systematic Review and Meta-analysis

An electronic search of the literature was performed interrogating the following databases: Cochrane Oral Health’s Trials Register, Cochrane Pregnancy and Childbirth’s Trials Register, Cochrane Central Register of Controlled Trials (CENTRAL), MEDLINE Ovid, Embase Ovid, LILACS BIREME Virtual Health Library (Latin American and Caribbean Health Science Information database), and US National Institutes of Health Ongoing Trials Register (ClinicalTrials.gov) and the WHO International Clinical Trials Registry Platform using a search string that was created combining appropriate keywords with the use of Boolean operators “AND” and “OR”. The search string for Cochrane Pregnancy and Childbirth’s Trials Register will be: periodont* or (scal* and polish*) or (root* and plan*) OR (tooth and scal*) or (teeth and scal*) or (dental and scal*) or “oral hygiene” or “oral health” or gingivitis.

Types of studies to be included: Randomized controlled trials

© 2022 Merchant AT et al. *JAMA Network Open.*

eFigure 1. Risk of Bias Chart Using the ROB 2 Tool

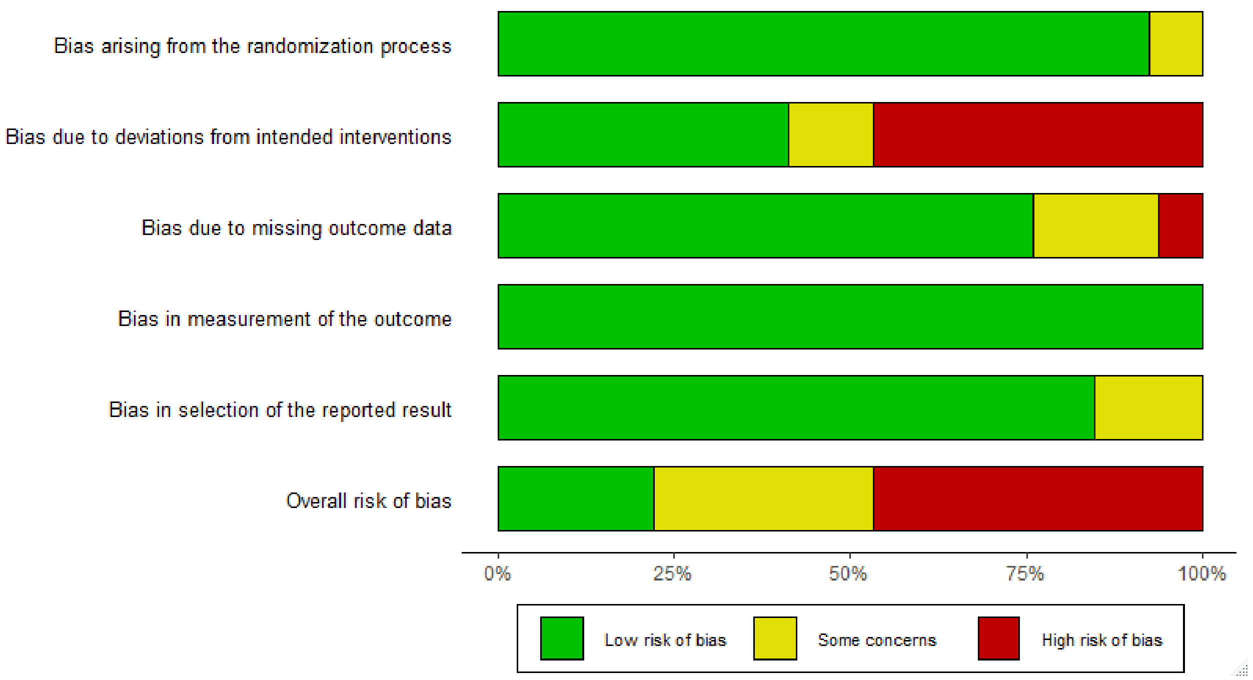

© 2022 Merchant AT et al. *JAMA Network Open.*

eFigure 2. Funnel Plot of Studies Included in Systematic Review and Meta-analysis

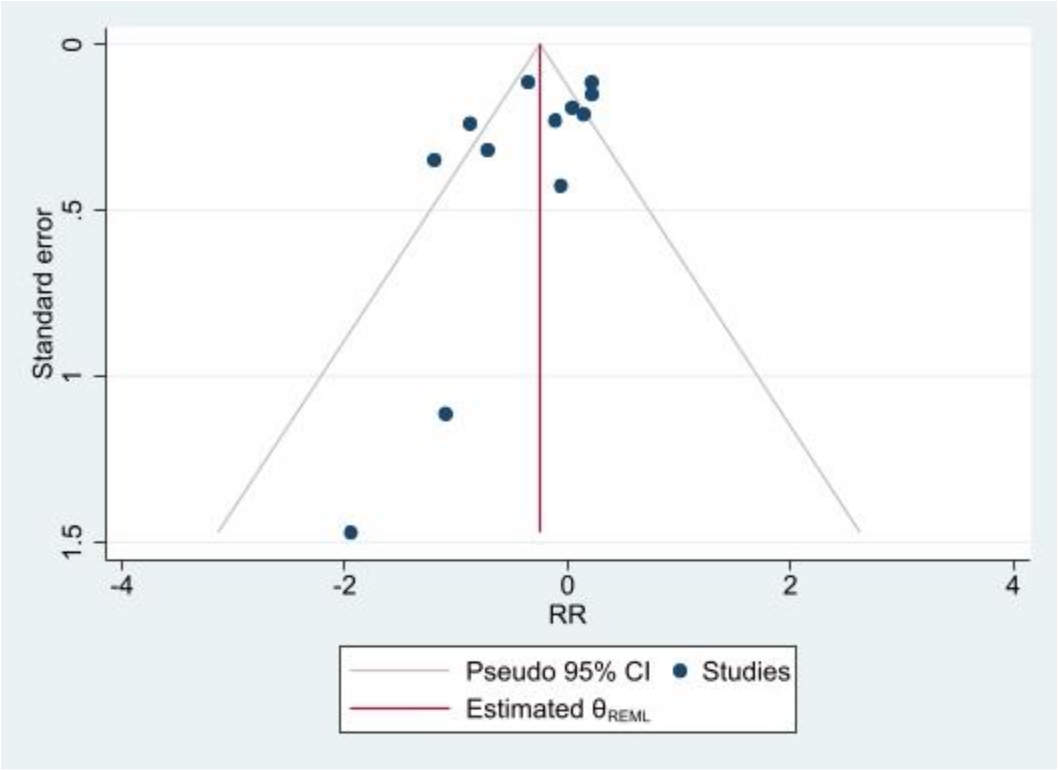

© 2022 Merchant AT et al. *JAMA Network Open.*

eFigure 3. Analyses for Preterm Birth Repeated Using Definition in Cochrane Review

Study	Treatment Yes	Treatment No	Control Yes	Control No	RR with 95% CI	Weight (%)
**Chlorhexidine**						
Lopez 2002	7	169	17	179	0.46 [0.19, 1.08]	4.78
Lopez 2005	18	552	17	269	0.53 [0.28, 1.02]	6.91
Newnham 2009	52	486	50	490	1.04 [0.72, 1.51]	11.56
Sadatmansouri 2006	0	15	3	12	0.14 [0.01, 2.55]	0.57
Tarannum 2007	53	48	68	23	0.70 [0.56, 0.88]	14.60
Heterogeneity: $\tau^2 = 0.05$, $I^2 = 45.65\%$, $H^2 = 1.84$					0.71 [0.51, 0.98]	
Test of $\theta_i = \theta_j$: Q(4) = 7.00, p = 0.14						
Test of $\theta = 0$: z = -2.11, p = 0.03						
**No Chlorhexidine**						
Farrell 2003	10	92	10	86	0.94 [0.41, 2.16]	4.99
Herera 2009	26	2	24	8	1.24 [0.99, 1.55]	14.52
Michalowicz 2006	44	363	38	367	1.15 [0.76, 1.74]	10.72
Offenbacher 2009	91	783	73	798	1.24 [0.93, 1.67]	13.13
Olivera 2011	27	91	31	90	0.89 [0.57, 1.40]	10.00
Radnai 2009	10	33	22	24	0.49 [0.26, 0.91]	7.25
Caneiro-Queija 2019	1	19	3	17	0.33 [0.04, 2.94]	0.97
Heterogeneity: $\tau^2 = 0.04$, $I^2 = 44.70\%$, $H^2 = 1.81$					1.03 [0.82, 1.29]	
Test of $\theta_i = \theta_j$: Q(6) = 10.51, p = 0.10						
Test of $\theta = 0$: z = 0.23, p = 0.82						
**Overall**					0.87 [0.70, 1.08]	
Heterogeneity: $\tau^2 = 0.07$, $I^2 = 62.47\%$, $H^2 = 2.66$						
Test of $\theta_i = \theta_j$: Q(11) = 29.76, p = 0.00						
Test of $\theta = 0$: z = -1.26, p = 0.21						
Test of group differences: $Q_b(1) = 3.42$, p = 0.06						

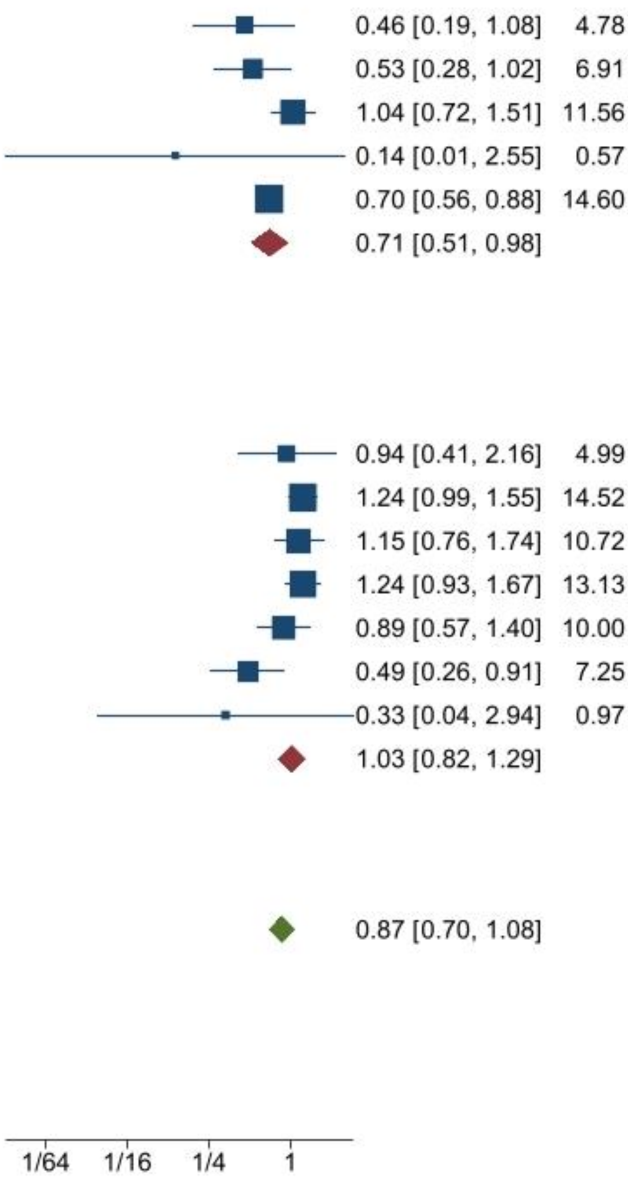

Random-effects REML model

© 2022 Merchant AT et al. *JAMA Network Open.*

eFigure 4. Leave-One-Out Meta-analyses for Preterm Birth

eFigure 5-A, Leave-one-out meta-analysis for chlorhexidine plus SRP group

```
Leave-one-out meta-analysis summary     Number of studies =      5
Random-effects model
Method: REML
```

Omitted study	RR	[95% conf.	interval]	p-value
Lopez 2002	0.657	0.405	1.067	0.090
Lopez 2005	0.599	0.315	1.138	0.117
Newnham 2009	0.465	0.285	0.758	0.002
Sadatmansouri 2006	0.581	0.348	0.967	0.037
Tarannum 2007	0.493	0.242	1.005	0.052
RR	0.558	0.337	0.925	0.024

eFigure 5-B, Leave-one-out meta-analysis for SRP only group

```
Leave-one-out meta-analysis summary     Number of studies =      7
Random-effects model
Method: REML
```

Omitted study	RR	[95% conf.	interval]	p-value
Farrell 2003	1.021	0.789	1.320	0.877
Herera 2009	0.939	0.693	1.273	0.685
Michalowicz 2006	0.966	0.709	1.316	0.824
Offenbacher 2009	0.944	0.696	1.280	0.711
Olivera 2011	1.032	0.779	1.368	0.826
Radnai 2009	1.165	1.002	1.355	0.047
Caneiro-Queija 2019	1.045	0.835	1.308	0.703
RR	1.027	0.816	1.293	0.818

© 2022 Merchant AT et al. *JAMA Network Open.*
