# Supplementary material for: Association of Chlorhexidine Use and Scaling and Root Planing With Birth Outcomes in Pregnant Individuals With Periodontitis: A Systematic Review and Meta-analysis
Source: JAMA Netw Open. 2022 Dec 19;5(12):e2247632. doi: 10.1001/jamanetworkopen.2022.47632 (PMC9856591; doi:10.1001/jamanetworkopen.2022.47632)
Supplement: Supplement 2. — Data Sharing Statement [file jamanetwopen-e2247632-s002.pdf]

Data Sharing Statement

Merchant. Association of Chlorhexidine Use and Scaling and Root Planing With Birth Outcomes in Pregnant Individuals With Periodontitis. *JAMA Netw Open*. Published December 19, 2022. doi:10.1001/jamanetworkopen.2022.47632

Data
Data available: Yes
Data types: Other (please specify)
Additional Information: We do not have access to individual level data as this was a meta-analysis
How to access data: Please email Anwar T. Merchant (merchant@mailbox.sc.edu) for data requests.
When available: With publication

Supporting Documents
Document types: None

Additional Information
Who can access the data: Researchers with approved proposals
Types of analyses: Not applicable
Mechanisms of data availability: Not applicable
